# Transcriptional Down-Regulation of Major Histocompatibility Complex as a Possible Pathogenesis for Meniere's Disease

**DOI:** 10.3389/fneur.2022.938740

**Published:** 2022-07-18

**Authors:** Kwang-Dong Choi, Eun Hye Oh, Hyun Sung Kim, Hyang-Sook Kim, Ji-Yun Park, Seo Young Choi, Jae-Hwan Choi

**Affiliations:** ^1^Department of Neurology, Pusan National University Hospital, Pusan National University School of Medicine and Biomedical Research Institute, Busan, South Korea; ^2^Department of Neurology, Pusan National University School of Medicine, Research Institute for Convergence of Biomedical Science and Technology, Pusan National University Yangsan Hospital, Yangsan, South Korea; ^3^Department of Neurology, Ulsan University Hospital, University of Ulsan College of Medicine, Ulsan, South Korea

**Keywords:** Meniere's disease, transcriptome analysis, immune system, MHC protein, differentially expressed gene

## Abstract

**Objectives:**

This study aimed to determine the underlying pathogenesis of Meniere's disease (MD) using transcriptome analysis.

**Methods:**

Total RNA was extracted from the peripheral blood mononuclear cells of 39 patients with MD and 39 controls. Through microarray analysis for nine patients and controls, the differentially expressed genes (DEGs) of those two groups were screened based on cut-off criteria (|fold changes| > 2.0 and adjusted *p*-value < 0.05). The functional enrichment analysis of DEGs was performed using Gene Ontology (GO).

**Results:**

There were 996 DEGs identified in the MD group: 415 were upregulated and 581 were downregulated. A functional enrichment analysis indicated that the downregulated DEGs were significantly enriched in terms related to immune system processes. Among them, 17 genes were enriched in terms for the major histocompatibility complex (MHC) protein complex, and the relative messenger RNA (mRNA) levels of three markedly downregulated DEGs [fold changes < −5: human leukocyte antigen (HLA)-DMA, HLA-DRB1, and HLA-DPB1] were significantly decreased in another 30 patients with MD compared with normal controls by quantitative reverse transcription-polymerase chain reaction (qRT-PCR). However, there were no correlations between the expression levels of these three genes and clinical data, such as age, onset age, time course, or hearing threshold.

**Conclusions:**

Our transcriptome analysis showed that the downregulated DEGs in MD were mainly associated with the immune system pathways including the MHC protein complex in MD. Remarkably, a breakdown in immunological tolerance mediated by MHC class II may contribute to the MD development, which has implications for targeted treatment.

## Introduction

Meniere's disease (MD) is a chronic disease of the inner ear characterized by episodic vertigo, fluctuating sensorineural hearing loss (SNHL) at low-to-medium frequencies, tinnitus, and aural fullness (1). Although endolymphatic hydrops (EH) is considered the histopathological hallmark of MD, the exact pathophysiology of MD is not yet fully understood. MD may be a multifactorial disorder associated with various etiologies, such as anatomical abnormalities, infections, and allergens. Autoimmunity has also been suggested as a potential cause of MD based on the increased prevalence of autoimmune diseases, the increased levels of autoantibodies, and the association with human leukocyte antigen (HLA) types in patients with MD (2–11). Since familial MD has been identified in 8−10% of cases, genetic factors might contribute to the MD development (2, 3, 12, 13). Next-generation sequencing and association studies using genomic DNA have revealed that numerous genes related to inner ear development, the immune-associated process, and systemic hemostatic derangement are associated with a susceptibility to MD (14–17). Polygenic multiallelic hypothesis has recently been proposed to explain the variable phenotype of MD (18). However, the genetic contribution to MD is still unclear due to the lack of a main validated gene across different populations.

Contrary to genome studies, transcriptome analysis using mRNA can establish the global pattern of gene expression and determine the biological functions of differentially expressed genes (DEGs) between two or more conditions (19). Therefore, transcriptome analysis has been widely used to identify the molecular pathogenesis and therapeutic targets in various malignancies and autoimmune diseases (20–22). However, only two previous studies have applied transcriptome analysis to MD (23, 24). One study using RNA sequencing found several DEGs associated with innate immune cells and autoimmune diseases in sporadic MD, suggesting that immune factors are involved in MD pathogenesis *via* regulation of immune system activities (23). Another study applied gene expression profiling for the differential diagnosis of MD and vestibular migraine, which revealed that the two disorders have different pro-inflammatory signatures (24).

This study aimed to provide novel insights into the pathogenesis and targeted treatment of MD using transcriptome analysis.

## Materials and Methods

We recruited 39 patients with definite MD according to the diagnostic criteria established by the Classification Committee of the Barany Society (1). The patients included 21 men and 18 women with an age ranging from 46 to 81 years (mean ± SD = 64.7 ± 8.1 years). The experiment has been conducted through the two-step process. Initially, the DEGs were screened using microarray analysis between nine patients with MD and normal controls. Then, the expression levels of selected DEGs were validated using quantitative reverse transcription-polymerase chain reaction (qRT-PCR) from other 30 patients with MD.

### RNA Extraction and Microarray Analysis

As described previously (23), total RNA was extracted from peripheral blood mononuclear cells (PBMCs) of nine patients with MD and nine controls without any vertigo or hearing loss using the TRIzol Reagent. The purity and quantity of RNA were measured using a spectrophotometer (NanoDrop ND-1000, Thermo Fisher Scientific, Wilmington, DE, USA), and the quality of RNA was further measured using a bioanalyzer (Agilent 2100, Agilent Technologies).

Isolated total RNA was amplified, labeled, and hybridized using the Affymetrix Human Clariom S Assay according to the manufacturer's protocol. In brief, the purified RNA sample was transcribed to double-strand cDNA using the GeneChip Whole Transcript (WT) amplification kit. That cDNA was then fragmented and biotin-labeled with terminal deoxynucleotidyl transferase (TdT) using the GeneChip WT terminal labeling kit. Approximately 5.5 μg of the labeled cDNA was hybridized onto the Affymetrix GeneChip Array at 45°C for 16 h. After washing, the arrays were stained on the GeneChip Fluidics Station 450 and scanned using the GeneChip Scanner 3000. Raw data were analyzed using the Affymetrix GeneChip Command Console software.

### Identification of DEGs and Functional Enrichment Analysis

To verify reproducibility between samples, Pearson's correlation test and multidimensional scaling (MDS) were performed. Raw data were summarized and normalized using the Robust Multi-array Average module implemented in Affymetrix Power Tools. The DEGs between MD and normal control samples were screened based on fold-change filtering combined with Student's *t*-tests. The false discovery rate (FDR) was controlled by the adjusted *p*-value using the Benjamini-Hochberg algorithm. Genes were considered to be differentially expressed when they conformed to the criteria of |fold changes| > 2 and adjusted *p*-value < 0.05.

To explain functional annotation for DEGs, the Gene Ontology (GO) enrichment analysis was performed using g:Profiler (https://biit.cs.ut.ee/gprofiler/). GO is a bioinformatics analysis that provides current scientific knowledge about the functions of genes and gene products and covers three biological terms: biological process (BP), cellular component (CC), and molecular function (MF). An adjusted *p*-value (FDR) < 0.05 was used as the criterion for statistical significance.

### Quantitative Reverse Transcription-Polymerase Chain Reaction (qRT-PCR)

The validation of selected DEGs was performed using qRT-PCR. Total RNA was extracted from PBMCs of other 30 patients with MD and healthy normal controls using the TRIzol Reagent (Life Technologies). Approximately 1 μg of total mRNA was reverse-transcribed into cDNA using M-MLV (Promega) according to the manufacturer's instructions. Gene expression was examined using qRT-PCR with the SYBR green PCR master mix and Rotor-Gene Q (both QIAGEN). The PCR conditions were as follows: initial denaturation at 95°C for 5 min, followed by 40 cycles of denaturation at 95°C for 15 s, amplification at 60°C for 30 s, and extension at 72°C for 30 s. Glyceraldehyde 3-phosphate dehydrogenase (GAPDH) was used as the reference for PCR normalization, and all PCRs were performed in triplicate. The relative expression levels of genes were calculated using the 2^−Δ*ΔCt*^ method.

### Statistical Analysis

Statistical analyses were conducted using SPSS software (version 22, SPSS, Chicago, IL, USA) for the clinical data and relative gene expression levels. Between microarray analysis (*n* = 9) and qRT-PCR (*n* = 30) groups, continuous (age, age at onset, time course, and hearing threshold) and categorical (gender, family history, delayed MD, headache, migraine, autoimmune disease, hearing stage, and steroid treatment) variables were compared using the Mann–Whitney test or Fisher's exact test. The relative gene expression levels obtained from qRT-PCR between MD and normal control samples were compared using Student's *t*-test, and the correlation between the clinical data and relative gene expression levels were analyzed using Pearson's correlation coefficient. The significance criterion was set at *p* < 0.05.

## Results

### Demographic and Clinical Characteristics

Detailed demographic and clinical characteristics of the patients are described in Table 1. The mean age at onset and time course was 57.5 ± 8.1 and 7.5 ± 4.3 years, respectively. It was found that three patients (7.7%) had a family history of episodic vertigo or SNHL. In total, 11 patients (28.2%) experienced a headache during attacks, but only one patient had migraine-type headache. In addition, four patients (10.3%) had a history of autoimmune diseases, such as rheumatoid arthritis (*n* = 2), inflammatory bowel disease (*n* = 1), and autoimmune hypothyroidism (*n* = 1). Nine patients (23.1%) had taken oral prednisolone or intratympanic dexamethasone injection to relieve vertigo attacks, all of whom showed a good response. There were no significant differences in demographic and clinical characteristics between the microarray analysis group (*n* = 9) and the qRT-PCR group (*n* = 30).

**Table 1:** Demographic and clinical characteristics of patients with the Meniere's disease (MD).

	**Total**	**Microarray group**	**qRT-PCR group**	***p*-value**
	**(*n* = 39)**	**(*n* = 9)**	**(*n* = 30)**	
Age, years, mean (SD)	65.0 (7.9)	67.1 (7.2)	64.7 (8.1)	0.379
Gender, female, *n* (%)	18 (46.2)	4 (44.4)	14 (46.7)	1.000
Age at onset, years, mean (SD)	57.5 (8.1)	59.0 (6.3)	57.3 (8.2)	0.436
Time course, years, mean (SD)	7.5 (4.3)	8.1 (5.4)	7.4 (4.1)	0.711
Family history, *n* (%)	3 (7.7)	1 (11.1)	2 (6.7)	0.556
Hearing loss at diagnosis, dB, mean (SD)	49.0 (18.1)	49.0 (21.9)	48.6 (17.7)	0.857
Delayed Meniere's disease, *n* (%)	5 (12.8)	1 (11.1)	4 (13.3)	1.000
Headache, *n* (%)	11 (28.2)	4 (44.4)	7 (23.3)	0.238
Migraine, *n* (%)	1 (2.6)	0 (0)	1 (3.3)	1.000
Autoimmune disease, *n* (%)	4 (10.3)	1 (11.1)	3 (10.0)	1.000
Hearing stage, *n* (%)
1	5 (12.8)	2 (22.2)	3 (10.0)	0.572
2	9 (23.1)	1 (11.1)	8 (26.7)	0.654
3	20 (51.3)	4 (44.4)	16 (53.3)	0.716
4	5 (12.8)	2 (22.2)	3 (10.0)	0.572
Steroid treatment	9 (23.1)	3 (33.3)	6 (20.0)	0.406

### DEG Identification by Microarray Analysis

**Figure 1:**
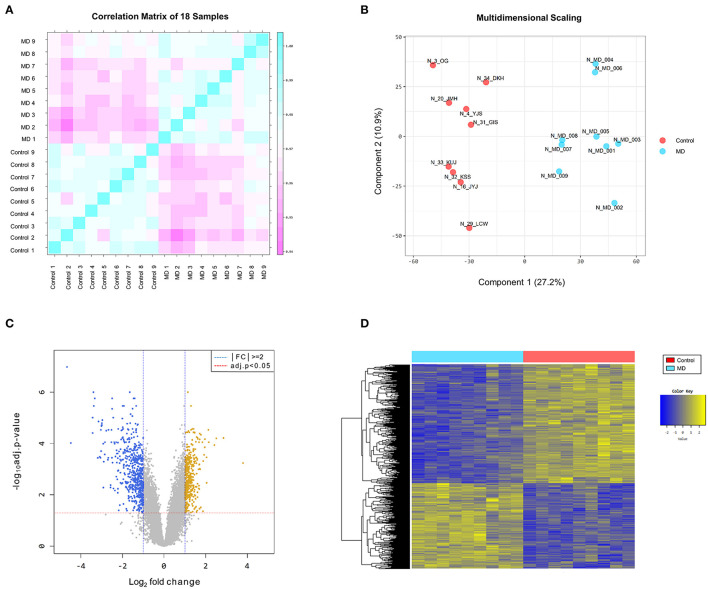
**(A)** Pearson's correlation analysis of samples. The color reflects the intensity of the correlation. The closer the correlation coefficient is to 1, the more it represents the blue color. There are strong correlations among the samples in the Meniere's disease (MD) and control groups. **(B)** Multidimensional scaling of samples. There is a great difference in gene expression patterns between the MD (blue color) and control (red color) groups. **(C)** The volcano plot of differentially expressed genes (DEGs). Based on the cut-off criteria (|fold changes| > 2.0 and adjusted *p*-value < 0.05), 996 DEGs are identified between the MD and control samples, comprising 415 upregulated (yellow dots) and 581 downregulated (blue dots) in the MD group. **(D)** The results of a hierarchical clustering analysis of upregulated and downregulated DEGs between the MD and control groups. Each group includes nine individuals and each column represents one individual's sample. The yellow and blue colors indicate high and low relative expression levels, respectively.

The reproducibility tests using Pearson's correlation test and MDS showed that there were positive correlations among the samples in the MD and control groups, and there was a great difference in gene expression patterns between the two groups (Figures 1A,B). These results indicate that the homogeneity of samples in the MD group and the heterogeneity of samples between the two groups.

The DEGs between the MD and control groups are depicted on the volcano plot in Figure 1C. Hierarchical cluster analysis of the upregulated and downregulated DEGs revealed distinctive expression patterns between them (Figure 1D). Based on the cut-off criteria (|fold changes| > 2 and adjusted *p*-value < 0.05), a total of 996 DEGs were detected between the MD and control samples, comprising 415 upregulated DEGs and 581 downregulated DEGs in the MD group (Supplementary Table 1). However, the number of DEGs with |fold changes| > 3 was considerably larger in the down-regulated DEGs than in the up-regulated DEGs (169 vs. 36). Furthermore, there were 41 genes with a distinct expression level (|fold changes| > 5) among the downregulated DEGs but only four among the upregulated DEGs.

### Functional Enrichment Analysis of DEGs

**Figure 2:**
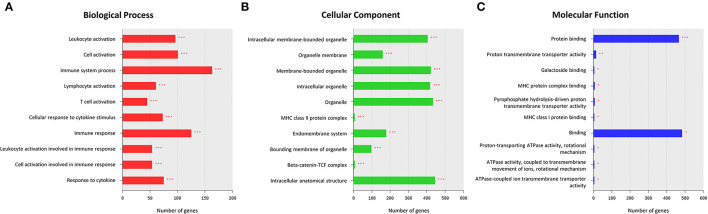
Results of a Gene Ontology (GO) enrichment analysis of downregulated differentially expressed genes (DEGs) in the MD group. The top enriched GO terms for the biological process (BP) **(A)**, cellular component (CC) **(B)**, and molecular function (MF) **(C)** are shown. The *x*-axis represents the number of genes enriched in each GO term. The *y*-axis represents GO terms with adjusted *p*-values of < 0.05 (^*^), < 0.01 (^**^), and < 0.001 (^***^).

The GO enrichment analysis was performed on all DEGs to identify their underlying functions. The upregulated DEGs were mostly enriched in BP terms for cellular localization and cellular metabolic processes, in CC terms for intracellular anatomical structure, and in MF terms for binding (Supplementary Figure 1). In contrast, most of the downregulated enrichments were related to BP terms for immune system processes, such as leukocyte activation, lymphocyte activation, and cellular response to cytokine stimulus (Figure 2A). For CC and MF, the downregulated DEGs were also enriched in terms related to the immune system processes, such as the major histocompatibility complex (MHC) class II protein complex, beta-catenin-TCF complex, MHC protein complex binding, and MHC class I protein binding (Figures 2B,C).

### Validation of Downregulated DEGs by qRT-PCR

**Figure 3:**
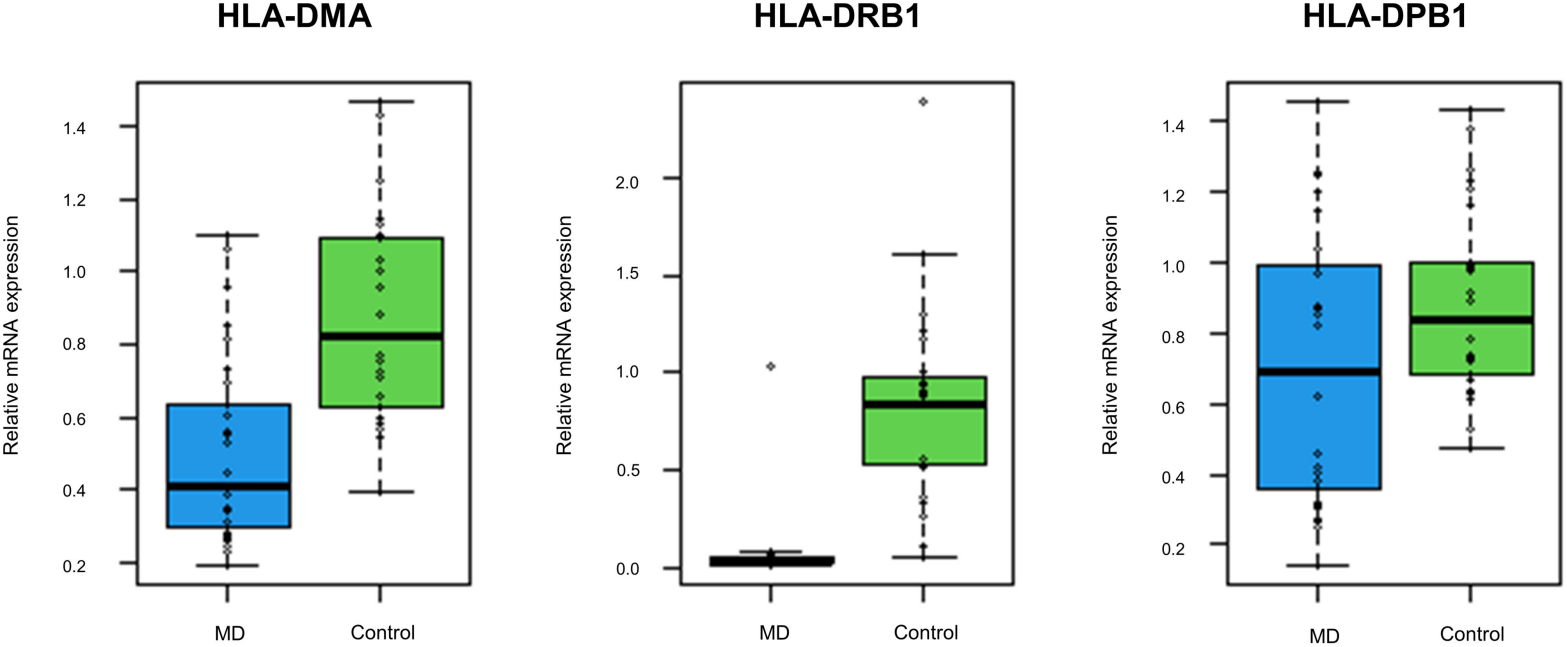
The expression levels of the top three downregulated differentially expressed genes (DEGs) associated with the MHC protein complex using quantitative reverse transcription-polymerase chain reaction (qRT-PCR). Compared with normal controls, the mRNA expression levels of human leukocyte antigen (HLA)-DMA, HLA-DRB1, and HLA-DPB1 are significantly decreased in 30 validation cohort with the MD (*p* < 0.001, *p* < 0.001, and *p* = 0.026, respectively).

The GO enrichment analysis indicated that most upregulated DEGs were enriched in non-specific terms, whereas the downregulated DEGs were mostly enriched in terms for immune system processes. Among them, specific terms associated with the MHC protein complex were deemed to be of particular interest (Table 2) since different HLA gene alleles have been reported among patients with MD (6–11). In total, 17 genes were enriched in terms for MHC protein complex (Table 3), and the three markedly downregulated DEGs (fold changes < −5: HLA-DMA, HLA-DRB1, and HLA-DPB1) were analyzed to verify their expression levels using qRT-PCR. The primer sequences used in the PCR analysis are listed in Supplementary Table 2. As shown in Figure 3, the relative mRNA expression levels of HLA-DMA, HLA-DRB1, and HLA-DPB1 were significantly decreased in 30 patients with MD compared with normal controls, which was consistent with the microarray data. However, there were no correlations between the expression levels of the three genes and clinical data, such as age, onset age, time course, or hearing threshold (Table 4).

**Table 2:** Gene Ontology (GO) terms associated with the major histocompatibility complex (MHC) protein complex in downregulated differentially expressed genes (DEGs).

**GO term**	**Specific term**	***p*-value**	**Down-regulated DEGs**
BP	GO:0002396-MHC protein complex assembly	3.4186e-05	*HLA-DMA, HLA-DRB1, HLA-DMB, TAPBPL, TAPBP*
	GO:0002501-peptide antigen assembly with MHC protein complex	0.00042583	*HLA-DMA, HLA-DRB1, HLA-DMB, TAPBPL*
	GO:0002399-MHC class II protein complex assembly	0.00277812	*HLA-DMA, HLA-DRB1, HLA-DMB*
	GO:0002503-peptide antigen assembly with MHC class II protein complex	0.00277812	*HLA-DMA, HLA-DRB1, HLA-DMB*
	GO:0019886-antigen processing and presentation of exogenous peptide antigen *via* MHC class II	0.00838024	*HLA-DMA, HLA-DRB1, HLA-DPB1, HLA-DMB, HLA-DOA, HLA-DPA1, FCER1G, CTSD, HLA-DQB1, DCTN3*
	GO:0002495-antigen processing and presentation of peptide antigen *via* MHC class II	0.01050122	*HLA-DMA, HLA-DRB1, HLA-DPB1, HLA-DMB, HLA-DOA, HLA-DPA1, FCER1G, CTSD, HLA-DQB1, DCTN3*
	GO:0002504-antigen processing and presentation of peptide or polysaccharide antigen *via* MHC class II	0.0127228	*HLA-DMA, HLA-DRB1, HLA-DPB1, HLA-DMB, HLA-DOA, HLA-DPA1, FCER1G, CTSD, HLA-DQB1, DCTN3*
	GO-2001190-positive regulation of T cell activation *via* T cell receptor contact with antigen bound to MHC molecule on antigen presenting cell	0.03779741	*LGALS9, HLA-DMB*
	GO-0002397-MHC class I protein complex assembly	0.03779741	*TAPBP, TAPBL*
CC	GO:0042613-MHC class II protein complex	1.0895e-05	*HLA-DMA, HLA-DRB1, HLA-DPB1, HLA-DMB, HLA-DOA, HLA-DPA1, HLA-DQB1*
	GO:0042611-MHC protein complex	0.00012583	*HLA-DMA, HLA-DRB1, HLA-DPB1, HLA-DMB, HLA-DOA, HLA-DPA1, HLA-DQB1*
MF	GO:0023023-MHC protein complex binding	0.01041392	*HLA-DMA, HLA-DRB1, HLA-DOA, LILRB2, HLA-DMB, TAPBPL*
	GO:0042288-MHC class I protein binding	0.02513155	*TAPBP, LILRB2, TUBB, PILRA, PILRB*

*BP, biological process; CC, cellular component; DEG, differentially expressed gene; GO, gene ontology; MF, molecular function; MHC, major histocompatibility complex*.

**Table 3:** Downregulated DEGs associated with the MHC protein complex.

**Gene**	**Transcript ID**	**Fold change**	**Adjusted *p*-value**
*HLA-DMA*	NM_006120	−7.819839	1.22843e-05
*HLA-DRB1*	NM_001243965	−6.434097	0.000362027
*HLA-DPB1*	NM_002121	−5.561917	0.000559052
*LGALS9*	NM_002308	−4.679215	0.00825859
*TAPBPL*	NM_018009	−3.642073	0.00018587
*CTSD*	NM_001909	−3.184350	0.00150167
*FCER1G*	NM_004106	−3.173098	0.00031412
*HLA-DQB1*	NM_001243961	−2.684463	0.01759631
*LILRB2*	NM_001080978	−2.619737	0.00048716
*HLA-DMB*	ENST00000414017	−2.599851	0.00010508
*HLA-DPA1*	NM_001242525	−2.528845	0.02204052
*TAPBP*	NM_003190	−2.486889	0.00909216
*PILRA*	NM_013439	−2.150609	0.00871555
*DCTN3*	NM_001281425	−2.119261	0.00042982
*HLA-DOA*	NM_002119	−2.106123	0.00434074
*PILRB*	NM_178238	−2.059781	0.00976276
*TUBB*	NM_001293214	−2.030527	0.00942943

*DEG, differentially expressed gene; MHC, major histocompatibility complex*.

**Table 4:** The correlation analysis between gene expression levels and clinical characteristics.

	***HLA-DMA***	***HLA-DRB1***	***HLA-DPB1***
Age	*r* = −0.007, *p* = 0.972	*r* = 0.025, *p* = 1.000	*r* = 0.145, *p* = 0.454
Onset age	*r* = −0.147, *p* = 0.446	*r* = −0.012, *p* = 0.951	*r* = 0.020, *p* = 0.920
Time course	*r* = 0.313, *p* = 0.099	*r* = 0.247, *p* = 0.196	*r* = 0.211. *p* = 0.272
PTA threshold	*r* = 0.202, *p* = 0.293	*r* = 0.242, *p* = 0.205	*r* = 0.156, *p* = 0.419

*PTA, pure tone audiometry*.

## Discussion

Our transcriptome analysis revealed about 1,000 genes that were expressed differentially between the MD and control samples, comprising 415 upregulated and 581 downregulated genes in the MD group. However, the number of genes with markedly different expression levels was considerably larger in the downregulated than in the upregulated DEGs, the former being mostly enriched in terms of immune system processes, such as MHC class II according to the bioinformatics analysis. Furthermore, qRT-PCR indicated that the expression levels of the three genes associated with MHC class II (HLA-DMA, HLA-DRB1, and HLA-DPB1) were significantly decreased in the validation cohort. These findings highlight a role of MHC class II in the immune system for MD pathogenesis.

In humans, the MHC class II protein complex is encoded by the HLA gene complex, such as HLA-DP, -DQ, -DR, -DM, and -DO (25). The MHC class II molecules are usually found on antigen-presenting cells, such as dendritic cells, macrophages, and B cells, and they play an important role in the immune system by presenting foreign antigens to CD4^+^ T cells. This process is not only significant for the immune responses protecting against invading pathogens but also for self-tolerance maintenance (26). Indeed, specific allele polymorphisms of HLA genes have been found to be associated with susceptibility or resistance to various autoimmune diseases, such as rheumatoid arthritis, autoimmune hepatitis, and type 1 diabetes mellitus (27–29). Autoimmunity seems to be involved in the pathogenesis of some inner ear diseases, such as rapidly progressive bilateral SNHL and autoimmune inner ear disease. Similarly, it was speculated that MD might be associated with autoimmunity or immune-mediated process based on the increased prevalence of autoimmune diseases, the increased levels of autoantibodies, and the positive response to steroid therapy in MD (2–5). Among the included patients in the present study, some also had concurrent autoimmune diseases and showed a good response to steroid therapy.

Previous studies have investigated the association between HLA and MD and found that several HLA alleles, such as DRB1^*^1602, DRB1^*^0405, and DRB1^*^1101 were significantly increased or decreased in patients with MD (6–11). However, the results varied with the racial background, and the association was usually observed in specific groups of MD, such as DRB1^*^0405 in the anti-type II collagen antibody positive group and DRB1^*^1101 in bilateral MD (10, 11). Nevertheless, the association between HLA alleles and MD indicates that immunological alterations are involved with the MD pathogenesis. In the present study, many genes encoding MHC class II protein complex were downregulated in the MD group, which suggests that MHC class II offers resistance to MD development. It is still unclear whether this finding is directly related to MD pathogenesis or merely reflects an epiphenomenon caused by recurrent MD attacks. However, there was no obvious relationship between the expression levels of three major HLA genes and clinical data, such as age, onset age, time course, or hearing threshold, suggesting that the decreased expression levels of HLA genes were not caused by recurrent MD attacks. Perhaps, the downregulation of MHC class II protein complex would result in a breakdown in immunological tolerance to the underlying pathogens or self-antigens of MD. Several possible mechanisms could contribute to the downregulation of MHC class II protein complex, such as haplotype-specific variation, epigenetic mechanism, or environmental factors (26). Further studies are needed to investigate the role of the MHC class II protein complex in MD development.

The present study was subject to some potential limitations. First, we obtained gene expression profiling from the total RNA of peripheral PBMCs. This approach may not determine whether immunological alterations in peripheral blood levels greatly influence the initiation of specific immune responses in the inner ear. Thus, transcriptome analysis using endolymph in the inner ear could be a better method for identifying the underlying pathogenesis of MD. Second, we have verified expression levels of only three down-regulated DEGs by qRT-PCR. It is possible that there are discrepancies in the expression levels between microarray analysis and qRT-PCR. Thus, the expression levels of more DEGs should be validated using qRT-PCR for a large number of patients. However, the results of qRT-PCR for three DEGs were consistent with those of microarray analysis, and the reproducibility tests showed the homogeneity of samples in each (MD and control) group and the heterogeneity of samples between the two groups, suggesting that the intra-group data repeatability might be acceptable.

In conclusion, our transcriptome analysis identified the downregulated DEGs associated with the immune system pathway in MD. The findings of our bioinformatics analysis indicate that these DEGs are likely to contribute to MD development through a breakdown in immunological tolerance mediated by the MHC class II protein complex. The findings of the present study have meaningful implications for further biological investigations and targeted treatment for MD.

## Data Availability Statement

The datasets presented in this study can be found in online repositories. The name of the repository and accession number can be found below: National Center for Biotechnology Information (NCBI) Gene Expression Omnibus (GEO), https://www.ncbi.nlm.nih.gov/geo/query/acc.cgi?acc=GSE202657.

## Ethics Statement

All experiments followed the tenets of the Declaration of Helsinki, and informed consents were obtained after the nature and possible consequences of this study had been explained to the participants. This study was approved by the institutional review boards of Pusan National University Yangsan Hospital (04-2020-029).

## Author Contributions

K-DC conducted the experiments and interpretation of the data, and wrote the manuscript. EO, HK, J-YP, and SC contributed to the interpretation and analysis of data. H-SK performed the experiment. J-HC conducted the design and conceptualization of the study, interpretation of the data, and revised the manuscript. All authors contributed to the article and approved the submitted version.

## Funding

This research was supported by the Basic Science Research Program through the National Research Foundation of Korea funded by the Ministry of Education (NRF-2020R1I1A3071617).

## Conflict of Interest

The authors declare that the research was conducted in the absence of any commercial or financial relationships that could be construed as a potential conflict of interest.

## Publisher's Note

All claims expressed in this article are solely those of the authors and do not necessarily represent those of their affiliated organizations, or those of the publisher, the editors and the reviewers. Any product that may be evaluated in this article, or claim that may be made by its manufacturer, is not guaranteed or endorsed by the publisher.
