# Supplementary material for: Transcriptional Down-Regulation of Major Histocompatibility Complex as a Possible Pathogenesis for Meniere's Disease
Source: Front Neurol. 2022 Jul 18;13:938740. doi: 10.3389/fneur.2022.938740 (PMC9339969; doi:10.3389/fneur.2022.938740)
Supplement: Supplementary file 2 [file Table_2.DOCX]

**Supplementary Table 2**. Polymerase chain reaction primer sequences for *HLA-DMA, HLA-DRB1* and *HLA-DPB1*

| Gene | Forward sequence | Reverse sequence |
| --- | --- | --- |
| *HLA-DMA* | 5’-TATTGGGTACCCCGGAACG-3’ | 5’-GAAGAATCAGTCACCTGAGC-3’ |
| *HLA-DRB1* | 5’-GTCTGAATCTGCACAGAGC-3’ | 5’-CAGAGTGCCCTTTCTGATTC-3’ |
| *HLA-DPB1* | 5’-GAGGCACAGTCTGATTCTG-3’ | 5’-ATCCTCGTTGAACTTTCTTGC-3’ |
