# Supplementary figures and images for: Transcriptional Down-Regulation of Major Histocompatibility Complex as a Possible Pathogenesis for Meniere's Disease
Source: Front Neurol. 2022 Jul 18;13:938740. doi: 10.3389/fneur.2022.938740 (PMC9339969; doi:10.3389/fneur.2022.938740)

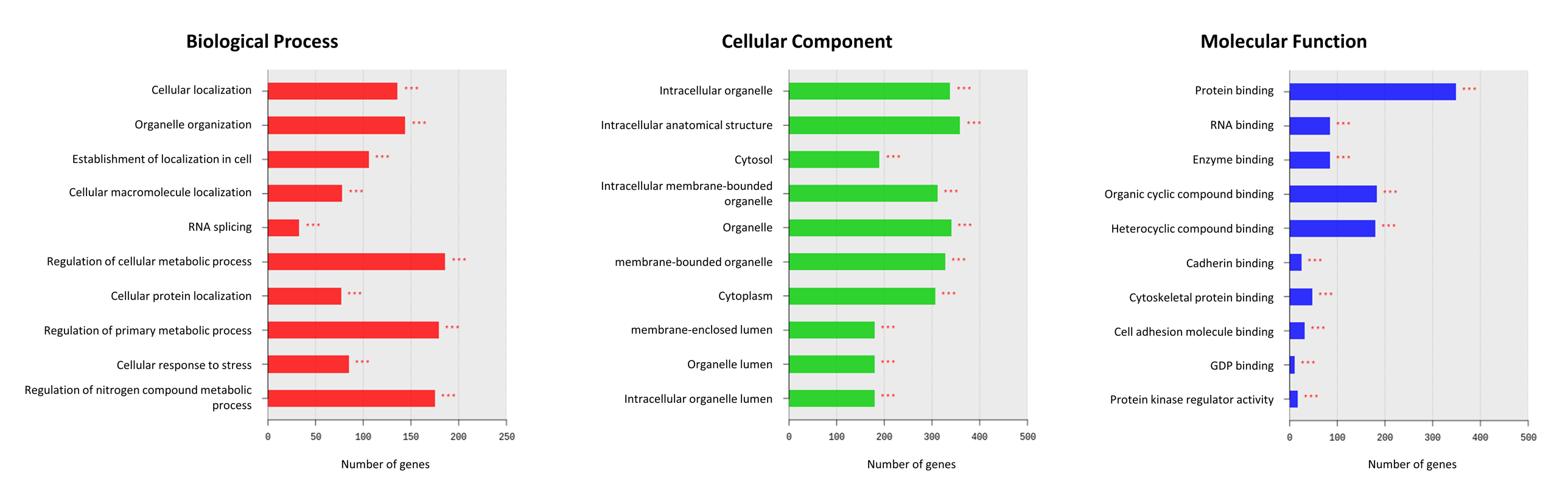

Supplement: Supplementary file 3 [file Image_1.TIF]
